# Interaction Effects of Social Isolation and Peripheral Work Position on Risk of Disability Pension: A Prospective Study of Swedish Women and Men

**DOI:** 10.1371/journal.pone.0130361

**Published:** 2015-06-23

**Authors:** Klas Gustafsson, Staffan Marklund, Gunnar Aronsson, Anders Wikman, Birgitta Floderus

**Affiliations:** 1 Department of Clinical Neuroscience, Division of Insurance Medicine, Karolinska Institutet, Stockholm, Sweden; 2 Department of Psychology, Stockholm University, Stockholm, Sweden; institute of Health Policy and Management, NETHERLANDS

## Abstract

**Purpose:**

The study examines various combinations of levels of social isolation in private life and peripheral work position as predictors of disability pension (DP). A second aim was to test the potential interaction effects (above additivity) of social isolation and peripheral work position on the future risk of DP, and to provide results for men and women by age.

**Method:**

The study was based on a sample of 45567 women and men from the Swedish population who had been interviewed between 1992 and 2007. Further information on DP and diagnoses was obtained from the Swedish Social Insurance Agency’s database (1993–2011). The studied predictors were related to DP using Cox’s proportional hazard regression. The analyses were stratified on sex and age (20–39 years, 40–64 years), with control for selected confounders.

**Results:**

Increased risks of DP were found for most combinations of social isolation and peripheral work position in all strata. The hazard ratios (HRs) for joint exposure to high degree of social isolation and a peripheral work position were particularly strong among men aged 20–39 (HR 5.70; CI 95% 3.74–8.69) and women aged 20–39 (HR 4.07; CI 2.99–5.56). An interaction effect from combined exposure was found for women in both age groups as well as a tendency in the same direction among young men. However, after confounder control the effects did not reach significance.

**Conclusions:**

Individuals who were socially isolated and in a peripheral work position had an increased risk of future DP. The fact that an interaction effect was found among women indicates that a combination of social isolation and peripheral work position may reinforce adverse health effects. There was no evidence that a peripheral work position can be compensated by a high degree of social intergration in private life.

## Introduction

Several factors in working life and in private life may be involved in the pathway of young adolescents from poor health to subsequent disability pension (DP). More knowledge about the causes of disability is highly relevant because the costs for DP and early retirement in Sweden have been very high during the last two decades. In this study, we will analyze aspects of social integration and labor market attachment–and the interaction between them.

In research on social integration, social isolation is measured either objectively in terms of not having social relations or not being integrated in a social group or subjectively in terms of perceived social isolation which concerns the feeling of loneliness [1]. Objective and subjective measures correlate but the overlap is not complete because objective social isolation is not always accompanied by a subjective negative affective response. Social isolation can, however, foster loneliness for those who interpret it as a reflection of their own limitations, particularly if the limitations are seen as immutable and not as a result of external conditions. According to Laursen and Hartl [1], social isolation often deprives the individual of tangible benefits provided by the group, whereas loneliness interferes with emotional, physical, and psychological performance. One research group which has explored loneliness and physiological pathways to morbidity and mortality concluded that physiological effects unfold over a relatively long time period [2–5]. In the case of depressive symptoms the results suggest a reciprocal influence as loneliness increases the risk of depression and depression increases the feelings of loneliness [6,7].

Similarly, the connection between objective social integration as well as subjective experienced loneliness and mortality was supported in a meta-analytic review from 2010 of 148 studies, indicating a 50% increased likelihood (weighted average effect size) of survival for participants with stronger social relationships [8]. The finding remained consistent across age, sex, initial health status, cause of death, and follow-up period. The association was stronger for complex measures of social integration (OR = 1.91; CI 1.63–2.23) than for binary indicators of residential status such as living alone versus living with others (OR = 1.19; CI 0.99–1.44). Perception of loneliness had an OR of 1.45; CI 1.08–1.94 for risk of mortality. The researchers concluded that the influence of social relationships on risk of mortality is comparable with other well-established risk factors for mortality. However, the average age at baseline in the studies was 63 years, and the generalizability of the results to younger age groups is unknown.

Against this background, it is reasonable to predict that several negative aspects of social integration may be associated with an increased risk of DP. There are only a few studies of high quality which have investigated this issue. In a large longitudinal Norwegian study involving a 10-year follow-up period and including a range of potential risk factors of DP [9], it was found that self-reported social isolation increased the hazard ratio (HR) of DP for men 20–49 years old (HR 1.80; CI 1.29–2.53) but not for older men and not at all for women. In women 20–49 years old, separation/divorce significantly increased the risk of DP but not for older women or for men [9].

In a series of Swedish prospective population studies, different aspects of social isolation and risk of DP have been investigated. In studies on young women, being a young lone mother (not married or cohabiting) was associated with increased risks of DP [10] and sickness absence [11]. Furthermore, a social integration measure was developed, based on country of birth, work status, family status, and social contacts with others [12]. This measure was shown to be a clear predictor of DP among women under 40 years of age, even after controlling for socioeconomic conditions and self-reported ill health. When type of DP diagnosis was considered, the effects of social isolation were mainly attributed to DP based on mental diagnoses and not to DP based on musculoskeletal diagnoses [13].

The second factor considered in this study has its background in the development of an increasingly heterogeneous workforce, where the traditional and simple dichotomy of employed versus unemployed has become too crude to reflect the complexity of labor markets and employment patterns in contemporary work life [14]. In the employment spectrum, between full-time employment with a permanent work contract and long-term unemployment, there is a large group of workers with different kinds of precarious employment working under uncertain, part-time, or temporary work contracts. Recent research has shown that there is a health gradient and health inequalities related to this core–periphery structure [14–16]. Several somewhat different methods have been used for placing individuals in this core–periphery structure but the results are similar in that the more peripheral work positions have higher risks of negative health outcomes. In a systematic review of temporary employment and various health outcomes, 27 articles were identified and critically appraised [17]. The researchers concluded that there was evidence for an association between temporary employment and psychological morbidity. Similarly, a recent Swedish longitudinal study found that health status in mid-life, particularly psychological distress, was associated with the patterns of an individual’s negative labor market experience, mainly independent of other social risk factors and previous health [18]. There seems to be only one study on work position and DP and that study showed an increased risk related to peripheral work position in the core–periphery continuum [19].

Working life participation or degree of labor market attachment may act as a strong social integrator, especially among young people. Most people with a full-time job and employment security are able to develop social and psychological bonds in the workplace which may counteract any potential effects of social isolation outside work. People with weak labor market attachment–being in a peripheral labor market position without income from work or with periods of unemployment–may find themselves in a socially isolated situation that may reinforce adverse health effects [20,21]. The stigmatization of being unemployed has been shown to increase the risk for social withdrawal [22,23].

Social isolation may also contribute to work life marginalization by cutting people off from useful information about employment opportunities [24]. Having a wide social network may improve the chances of employment. Individuals who are less attached to the labor market through part-time work or periods of non-work may under certain circumstances be able to compensate some of the negative effects through social networks and integration in the areas of private life [25].

The incidence of DP has increased in later years in Sweden, particularly in younger ages [26]. At the same time the level of unemployment has grown as well as the number of people in precarious employment positions. Increasing shares of the population are living alone and family building is gradually starting at higher ages [27].

In Sweden, DP can be granted if an individual aged 19–64 has been assessed to have a reduced work capacity due to a medically certified disease or injury. It can be granted full-time or part-time (25%, 50% or 75%) [26].

The main aim of the study was to examine different combinations of social isolation in private life, and peripheral work position as predictors of future DP. The first purpose was to estimate potential interactions between the two factors with regard to DP. The hypothesis was that individuals who were socially isolated and in a peripheral work position should have an excess risk compared to those who were unexposed to both factors. This excess should deviate from the sum of the excess in relative risks produced by the two factors occurring on their own. A surplus would indicate a synergistic interaction and, a departure from an additive effect. The methodology is commonly used in epidemiology and has been elaborated by Rothman [28]. A second purpose was to describe differences between women and men and between age groups in this respect.

## Methods

### Study population

The study population comprised 49161 men and women, 20 to 64 years old during follow-up, and born between 1928 and 1987, who were interviewed by Statistics Sweden between 1992 and 2007 using the Swedish Surveys of Living Conditions (SSLC) [29–32]. Covering a broad range of living conditions, these annual surveys were based on year-specific random samples of the population and were conducted through face-to-face interviews between1992 and 2005 and through telephone interviews in 2006 and 2007. The annual response rates (1992–2007) varied between 76% and 82%. If an individual happened to be included in more than one annual sample, data from the earliest year was used. Additional data on the study group were gathered from the Longitudinal Database for Health Insurance and Labor Market Studies (LISA) (1992–2011) and the Swedish Social Insurance Agency’s database Micro Data for Analysis of Social Insurance (MiDAS) (1993–2011). Men and women who had obtained a DP prior to being interviewed (n = 3594) were excluded from the study. Of the 45567 remaining individuals, 4376 were granted DP within the follow-up period (1993–2011).

The data from Statistics Sweden were based on informed consent; written information is given to the individuals and consent is obtained by answering the survey. Data held by the Social Security Authority about granted disability pensions are partly collected for research purposes without consent from the individual. The Swedish law on Research Ethics states that research using sensitive registry data has to be approved by the Regional Research Ethics committee. All data used in the study were de-identified by Statistics Sweden before they were made available to the research team. The study and the consent procedure was approved by the Regional Ethical Review Board in Stockholm in 2011, Sweden (Dnr: 2011/1689-31/5).

### Outcome variable

The category labelled “all DP” covered all DP cases granted in 1993–2011 (n = 4376). This included all diagnostic groups of the ICD-10 [33], categories A through Z, as well as the unspecified diagnoses (70 cases). These unspecified cases had received a DP in 1993 and were mainly a result of the fact that before 1994, individuals over 60 years of age could receive a DP partly due to labor market reasons. Although DPs could either be full time or part time (25%, 50%, or 75%), no distinction was made between full-time and partial DP in this study.

The population DP rate has varied between 8.3% in 1993, 10.7% in 2005 and 7.3% in 2011. The share of part-time DP cases has varied between 20% and 28% in the study period. A higher proportion of women than of men and a slightly higher proportion of younger individuals were granted part-time DP. A large share of the part-time DP cases are later transferred into full-time DP [34]. As this study has a long follow up period no separation between part-time and full-time DP was done. The data were obtained from the MiDAS database.

### Exposure variables

#### Social isolation

Three variables were used to create an index of social isolation (SI). The response categories of each variable were assigned values between 1 and 4 points (p). All items have been developed within the SSLC [29–32] and have been used in many studies [12,13,35].

- Family status: cohabiting with children (4p); cohabiting without children (3p); lone with children (2p); and lone without children (1p).
- Social contacts: “In general how often do you meet with friends, acquaintances or relatives? Do not include current neighbors or workmates.” The response scale was: several times a week (4p); about once a week (3p); about once a month (2p); and more seldom (1p).
- Having close friends: “Do you have one or more really close friends with whom you can get in contact and discuss all sorts of things? Do not include members of your family or your household. The response choices were: yes (4p) and no (1p).

The three social isolation variables and the distributions of the response alternatives are presented in Table 1. The points assigned to the individuals’ answers were added together to create the SI index. The index varied between 3p and 12p, and the distribution was trichotomized into groups classified as: “no social isolation” (10–12p, prevalence 41.52%), “partial social isolation” (8–9p, prevalence 40.57%), and “social isolation” (3–7p, prevalence 17.92%). Furthermore, the index was dichotomized with the cutoff close to the 20th percentile (17.92%), thereby selecting the most socially isolated (3–7p) with the remaining individuals as reference (8–12p). The dichotomy was chosen to balance between statistical power and exposure contrasts in the tests of interaction. It should be noted that social contacts were restricted to the private life arena, and did not include social interaction at work (colleagues, fellow workers, foremen, management, and others).

**Table 1:** Social isolation, peripheral work position and selected confounders.

	No disability pension				Disability pension			
	Men		Women		Men		Women	
Variable	n	%	n	%	n	%	n	%
**Social isolation**								
*Family status*								
Cohabiting with children (4p)	7816	37	7867	39	566	32	880	34
Cohabiting without children (3p)	5923	28	6331	32	642	36	983	38
Lone with children (2p)	397	2	1346	7	45	3	272	10
Lone without children (1p)	7029	33	4482	22	517	29	471	18
Missing	-		-		-		-	
*Social contact frequency*								
Several times a week (4p)	5784	27	4569	23	343	19	388	15
About once a week (3p)	6955	33	7181	36	524	30	841	32
About once a month (2p)	5921	28	6072	30	573	33	918	35
More seldom (1p)	2465	12	2171	11	321	18	450	17
Missing	40		33		9		9	
*Having close friends*								
Yes (4p)	16613	79	17965	90	1232	71	2206	85
No (1p)	4378	21	1975	10	505	29	384	15
Missing	174		86		33		16	
**Peripheral work position**								
*Employment income*								
Employed (3p)	17525	83	15898	79	1299	73	1950	75
Not employed with some income(2p)	2123	10	2376	12	195	11	244	9
Not employed with no income (1p)	1517	7	1752	9	276	16	412	16
Missing	-		-		-		-	
*Work hours*								
Full-time work (3p)	14114	67	9190	46	1079	61	1111	43
Part-time work (2p)	1235	6	5997	30	78	4	800	31
No work hours (1p)	5815	27	4837	24	612	35	694	27
Missing	1		2		1		1	
*Days of unemployment*								
No days (3p)	16976	80	16262	81	1286	73	1971	76
1–180 days (2p)	2160	10	2239	11	179	10	285	11
181– days (1p)	2029	10	1525	8	305	17	350	13
Missing	-		-		-		-	
**Confounders**								
*Age*								
20–39 years	11521	54	10940	55	419	24	810	31
40–64 years	9644	46	9086	45	1351	76	1796	69
Missing^1^	-		-		-		-	
*Country of birth*								
Born in Sweden with Swedish-born parents	17173	81	16004	80	1370	78	2013	77
Born in Sweden with one or both parents foreign born	1599	8	1509	8	77	4	164	6
Foreign born	2354	11	2478	12	319	18	421	16
Missing	39		35		4		8	
*Long-standing illness*								
No	14484	68	13334	67	627	35	907	35
Yes	6681	32	6692	33	1143	65	1699	65
Missing	-		-		-		-	
*Total*	21165	100	20026	100	1770	100	2606	100

Percentage distribution and number of men and women, by disability pension.

#### Peripheral work position

An individual’s position on the core–periphery scale was based on data from the SSLC surveys [29–32] and the LISA database [36,37]. The distance from the core was measured by three variables combined into an index of peripheral work position (PWP). The response categories of each variable were given a value between 1 and 3 points.

- Employment income. Data on personal income for the year of the SSCL interview were used to determine whether a person was to be considered employed or not. A procedure developed by Statistics Sweden for attaining a good match to other labor force statistics when assessing the employment status of different groups of individuals was followed [37]. Three categories were defined: employed with income (3p), not employed with some income (2p), and not employed with no income (1p).
- Work hours. Individuals categorized as employed may have been working to a limited extent through part-time employment, seasonal employment, or employment only during part of the year. Information about work hours was added to complement the employment income variable. The variable was based on the interview data on whether the person engaged in full-time work (3p), part-time work (2p), or had no work hours (1p) for the week preceding the SSLC interview.
- Days of unemployment. The number of registered days of unemployment was also added to complement the employment income variable. The data for this variable were sourced from the LISA database for the year of the SSLC interview. The categories used were: no days of unemployment (3p), 1–180 days (2p), and more than 180 days (1p).

The distributions for the response categories of the three variables are presented in Table 1.

The points attached to the individuals’ answers were added together to create the PWP index. The index varied between 3p and 9p, and was trichotomized into the following groups: “peripheral work position” (3–6p, prevalence 20.77%), “partly peripheral work position” (7–8p, prevalence 30.42%), and “non-peripheral work position” (9p, prevalence 48.80%). The index was also dichotomized with the cutoff close to the 20th percentile (20.77%). Individuals with a peripheral work position (3–6 p), were studied with the remaining individuals as reference (7–9 p).

Finally, the trichotomized indices for social isolation and peripheral work position were paired together to form nine combinations (Table 2). To test for potential interaction effects, the dichotomized categories were paired into four combinations (Table 2).

**Table 2:** Nine and four combinations of social isolation and peripheral work position.

No.	Variable	Label	n	%
	**Nine combinations**			
1	No social isolation, 10–12 p, and non-peripheral work position, 9 p ^a^	no SI + no PWP	9282	20.52
2	Partial social isolation, 8–9 p, and non-peripheral work position, 9 p	part SI + no PWP	8652	19.13
3	Social isolation, 3–7 p, and non-peripheral work position, 9 p	SI + no PWP	4187	9.26
4	No social isolation, 10–12 p, and partial peripheral work position, 7–8 p	no SI + part PWP	6404	14.16
5	Partial social isolation 8–9 p, and partial peripheral work position, 7–8 p	part SI + part PWP	5229	11.56
6	Social isolation, 3–7 p, and partial peripheral work position, 7–8 p	SI + part PWP	2135	4.72
7	No social isolation, 10–12 p, and peripheral work position 3–6 p	no SI + PWP	3090	6.83
8	Partial social isolation, 8–9 p, and peripheral work position, 3–6 p	part SI + PWP	4464	9.87
9	Social isolation, 3–7 p, and peripheral work position 3–6 p	SI + PWP	1781	3.94
	**Four combinations**			
1,2,4,5	No/partial social isolation, 8–12p, and no/partial peripheral work position, 7–9 p ^a^	no/part SI + no/part PWP	29567	65.38
3,6	Social isolation, 3–7 p, and no/partial peripheral work position, 7–9 p	SI + no/part PWP	6322	13.98
7,8	No/partial social isolation, 8–12p, and peripheral work position, 3–6 p	no/part SI + PWP	7554	16.70
9	Social isolation, 3–7 p, and peripheral work position, 3–6 p	SI + PWP	1781	3.94

^a^ Reference category

We used categorical data to build indices which could be more valid estimates of social isolation and peripheral work position than the separate original response categories. However, we do not want to claim that this has left us with continuous variables. Instead we have assessed the individuals in two ways: by a more detailed grouping (yielding 9 exposure groups), and a more crude dichotomization (yielding 4 exposure groups). This categorization shows that there is a rank order between the extreme categories, but the rank order between categories in the middle could not be known at the outset.

### Potential confounders

In addition to sex and age (one-year intervals), country of birth and long-standing illness were selected as potential confounding factors. The data were derived from the SSLC database [29–32]:

- Country of birth: born in Sweden with Swedish-born parents (reference); born in Sweden with one or both parents foreign born; or foreign born.

Educational level and private financial situation were also considered as potential confounders, but were not included as confounders in the final estimations. Both were tested and found to be associated with the risk of DP, but inclusion in the final model did not change the results to any considerable extent.

Since poor health may have an effect on the exposure variables, and may independently contribute to the risk of DP, there were reasons to control for the health status of individuals at the start of follow-up. The data on long-standing illness were obtained from the SSLC surveys.

- Self-reported long-standing illness was assessed by the open-ended question: “Do you have any chronic or long-term illness or health problem?” The questioning was carried out by a trained staff member at Statistics Sweden to provide a solid basis for coding according to the WHO International Classification of Disease, 8^th^ revision (ICD-8). In this study, the summary coding of yes/no (with “no” as reference) was used.

Information on sickness absence preceding DP was available. However, due to the fact that almost all DP cases had been on sickness absence and the strong correlation with self-reported long-standing illness this variable was not used as a confounder.

### Statistical analyses

The participants from the annual SSLC surveys from 1992 to 2007 were consecutively added to the cohort, and the follow-up for each sub cohort started the year after the interview (January 1, 1993–2008). As the interviews took place during January-March each year the “wash-out” period varied between 9 months and 12 months. About 70% of DP cases are decided within 4 months after application, which means that most cases did not have an ongoing process affecting the interview. The follow-up period for the participants ended on November 30, 2011, or the year they reached 64 years of age, went on DP, emigrated, went on preterm old-age pension, or died, whichever came first. The mean number of years of follow-up was twelve years (SD 5.0). All analyses were stratified on four groups: young men (aged 20–39), young women (aged 20–39), older men (aged 40–64), and older women (aged 40–64). All statistical analyses were conducted with SAS, version 9.4., statistical software (SAS Institute, Inc., Cary, North Carolina) using the PHREG procedure.

**Fig 1:**
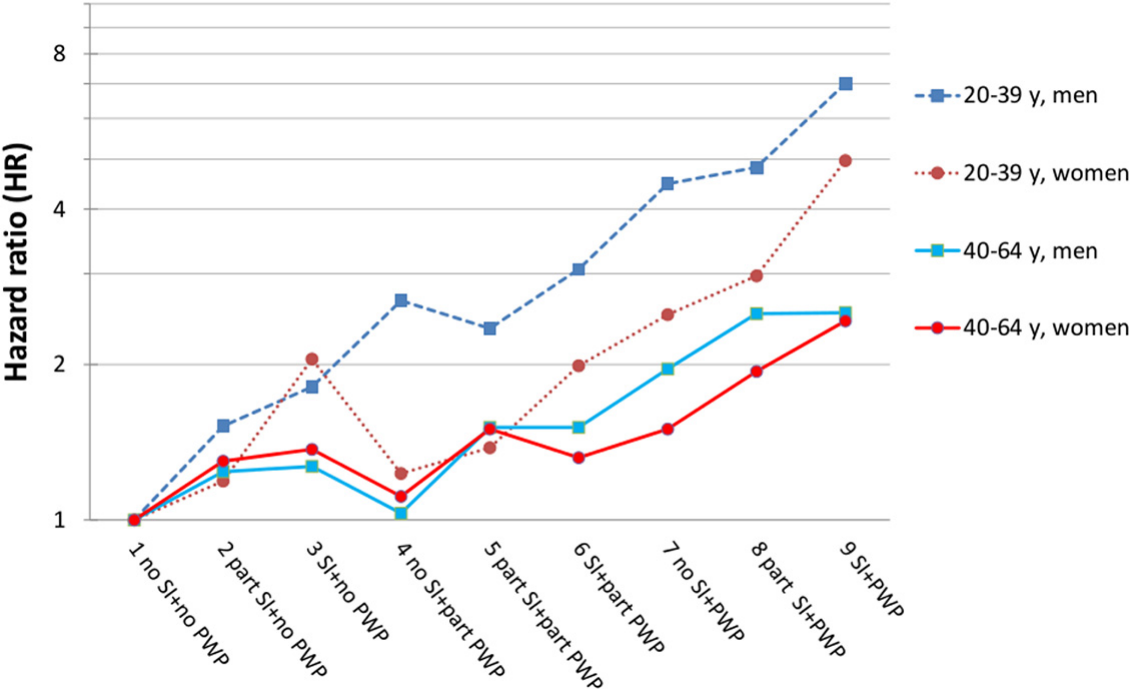
Nine categories classifying the co-exposure to social isolation (SI) and peripheral work position (PWP) related to the risk (HR) of disability pension. With adjustment for age at interview (one-year intervals) and year (y) of interview (log scale).

The analyses were conducted in two steps. First, the different combinations of social isolation and peripheral work position were related to DP, adjusting for age at interview (one-year intervals) and year of interview (Fig 1), followed by an extended adjustment also including country of birth and self-reported long-standing illness (Table 3). This was applied to both the nine-group and four-group categorizations of the indices of social isolation and peripheral work position. The hazard ratios (HRs) and 95% confidence intervals (CI) of being granted a DP were estimated using Cox’s proportional hazards regression analysis using the PHREG procedure. Proc life test was used to test violation of proportionality. First, we created graphs by means of the SAS proc life test (log cumulative hazard plots) and there were no violations of the proportionality in the visual assessment. Further, we used the statistic test statement, and there were no significant violations of the proportionality for women or among younger men. For older men the test suggested a significant departure from proportionality. However, in large samples statistically significant results (Kaplan-Meier estimates) are easily obtained also for deviations of minor/no importance.

**Table 3:** Co-exposure to social isolation and peripheral work position related to risk of disability pension (DP)^a^.

	Ages 20–39											Ages 40–64								
	Men (n = 11940)					Women (n = 11750)						Men (n = 10995)				Women (n = 10882)				
	P^b^	n^c^	HR^d^	95% CI		P^b^	n^c^	HR^d^	95% CI		P^b^	n^c^	HR^d^	95% CI		P^b^	n ^c^	HR^d^	95% CI	
DP, all diagnoses		407					801					1325					1785			
**Combinations of social isolation and peripheral work position** ^**e**^																				
1 no SI + no PWP	23	50	**1**			19	107	**1**			22	217	**1**			17	230	**1**		
2 part SI+ no PWP	21	55	**1.55**	1.06	2.28	12	67	1.10	0.81	1.50	22	271	**1.21**	1.01	1.44	22	376	**1.22**	1.03	1.44
3 SI + no PWP	8	33	**1.74**	1.12	2.70	3	30	**1.86**	1.24	2.79	19	240	**1.22**	1.02	1.47	8	149	**1.28**	1.04	1.58
4 no SI + part PWP	8	45	**2.62**	1.75	3.92	22	157	1.19	0.93	1.52	9	89	1.01	0.79	1.29	17	277	1.09	0.92	1.30
5 part SI + part PWP	11	32	**2.31**	1.47	3.62	12	69	1.26	0.92	1.71	8	111	**1.43**	1.14	1.81	15	298	**1.40**	1.18	1.67
6 SI + part PWP	3	19	**2.83**	1.67	4.81	3	28	**1.73**	1.14	2.62	7	100	**1.39**	1.10	1.77	6	112	1.16	0.93	1.46
7 no SI + PWP	5	45	**3.77**	2.50	5.67	13	151	**2.29**	1.78	2.94	4	74	**1.65**	1.26	2.15	5	101	1.25	0.98	1.58
8 part SI + PWP	15	86	**3.99**	2.76	5.76	13	120	**2.53**	1.93	3.32	5	118	**2.16**	1.72	2.71	6	146	**1.66**	1.34	2.05
9 SI + PWP	4	42	**5.70**	3.74	8.69	4	72	**4.07**	2.99	5.56	5	105	**2.11**	1.66	2.67	4	96	**1.86**	1.46	2.38

With control for potential confounding factors and stratification on age and sex.

^a^ All incident cases of DP, including unspecified DP diagnoses (n = 4376). Missing data (n = 343), with DP (n = 58) and without DP (n = 285).

^b^ Prevalence (P) of the exposure categories (%).

^c^ Number of cases (n).

^d^ Hazard ratio (HR) and 95% confidence interval (CI), adjusted for country of birth, self-reported long-standing illness

age at interview (one-year intervals), and year of interview.

^e^ Nine categories classifying the co-exposure to social isolation (SI) and peripheral work position (PWP). See method section.

Further, potential interaction effects of social isolation and peripheral work position on the risk of DP were tested. The assumption was that these two factors may interact in different ways, but our main hypothesis was that simultaneous presence of social isolation and peripheral work position should give a strengthened effect, above the added effect of the two factors acting on their own.

Interaction is possible to assess through linear regression models with multiplicative terms, but the variables has to be at least interval scales, which was not the case in our study. It can also be assessed by logistic regression with results on the multiplicative level, but this gives mainly an answer on statistical significance, and the results are not easily attributed to the common risk estimates.

However, it is also possible that social isolation and peripheral work position may both be required in the pathway towards risk of disability. This is sometimes called biological interaction [28,38–42]. Rothman has shown that risk factors of this kind adhere to an additive model [28]. Our main interest has been to explore potential synergistic effects between different levels of social isolation and peripheral work position relative to future disability pension, but there are also possibilities of antagonistic effects between the two exposure variables. Synergistic effect means interaction above additivity (R_11_ > R_01_+R_10_); antagonistic interaction can only occur if R_11_< R_01_+R_10_.

This choice of methodology is partly related to how we regarded the nature of the used measurements of social isolation and peripheral work position. Both were indexes based on questionnaire items where we could not assume that the distance between the measurement points were the same. To facilitate an interpretation the results were shown close to the risk estimates used. This was also the reason why we decided to present detailed information based on two different sets of combinations of the two main factors. To assess interaction the relative risks of the two factors acting alone were compared with the relative risk for joint exposure. The test statistic was the synergy index–S [38–41,43], with a 95% confidence interval (CI). The following formula was used:

$$•\;\text{S}=[{\text{HR}}_{11\left(\text{SI}+\text{PWP}\right)}-1]/[({\text{HR}}_{10\left(\text{SI}+\text{no}/\text{part PWP}\right)}-1)+({\text{HR}}_{01\left(\text{no}/\text{part SI}+\text{PWP}\right)}-1)].$$

S is a measure of the excess in relative risk, in this case the HR, from exposure to both social isolation and peripheral work position (SI + PWP) relative to the sum of the excess in relative risks shown for the two exposures when occurring one at a time (SI + no/part PWP and no/part SI + PWP, respectively). An S above 1 indicates the presence of an interaction above additivity (synergy), and an S lower than 1 indicates the presence of an antagonistic effect.

Individuals with missing data on one or more of the original items used to create an index were omitted (in all 343 out of 45567), and in the analyses of both indices combined, individuals with missing data on either index were omitted. This lowered the numbers slightly in the analyses of interaction (Tables 3 and 4).

**Table 4:** Co-exposure to social isolation and peripheral work position related to risk of disability pension ^a^.

	**Ages 20–39**															
	**Men** (n = 11940)								**Women** (n = 11750)							
	P^b^	n ^c^	HR^d^	95%	CI	HR^e^	95%	CI	P^b^	n ^c^	HR^d^	95%	CI	HR^e^	95%	CI
**Social isolation and peripheral work position** ^f^		407								801						
1 no SI+no PWP	64	182	1			**1**			66	400	**1**			**1**		
2 SI+no PWP	11	52	1.31	0.97	1.79	1.26	0.93	1.71	5	58	**1.69**	1.28.	2.23	**1.57**	1.19	2.07
3 no SI+PWP	20	131	**2.76**	2.20	3.43	**2.30**	1.82	2.91	25	271	**2.24**	1.91	2.62	**2.07**	1.76	2.43
4 SI+PWP	4	42	**4.17**	2.98	5.83	**3.36**	2.39	4.74	4	72	**4.12**	3.20	5.30	**3.43**	2.65	4.76
**Interaction**			S^d^			S^e^					S^d^			S^e^		
Synergy index (S)			1.53	0.93	2.51	1.51	0.87	2.65			**1.62**	1.07	2.44	1.50	0.96	2.33
	**Ages 40–64**															
	**Men** (n = 10995)								**Women** (n = 10882)							
	P^b^	n ^c^	HR^d^	95%	CI	HR^e^	95%	CI	P^b^	n ^c^	HR^d^	95%	CI	HR^e^	95%	CI
**Social isolation and peripheral work position** ^f^		1325								1785						
1 no SI+no PWP	61	688	**1**			**1**			71	1181	1			**1**		
2 SI+no PWP	25	340	1.12	0.98	1.28	1.09	0.96	1.24	15	261	1.09	0.95	1.25	1.03	0.90	1.18
3 no SI+PWP	9	192	**1.89**	1.61	2.22	**1.64**	1.40	1.93	11	247	**1.39**	1.39	1.60	**1.22**	1.06	1.41
4 SI+PWP	5	105	**2.09**	1.70	2.57	**1.77**	1.44	2.19	4	96	**1.92**	1.56	2.37	**1.54**	1.24	1.90
**Interaction**			S^d^			S^e^					S^d^			S^e^		
Synergy index (S)			1.08	0.67	1.74	1.06	0.60	1.88			**1.92**	1.01	3.64	2.16	0.76	5.86

Rothman’s synergy index (S) was calculated with 95% confidence intervals.

^a^ All incident cases of DP, including unspecified DP diagnoses (n = 4376).

^b^ Prevalence (P) of the exposure categories (%).

^c^ Number of cases (n).

^d^ Hazard ratio (HR), and Rothman’s synergy index (S), and 95% confidence interval (CI), adjusted for age at interview and year of interview.

^e^ Hazard ratio (HR), Rothman’s synergy index (S), and 95% confidence interval (CI), adjusted for country of birth, self-reported long-standing illness, age at interview (one-year intervals), and year of interview.

f: Four categories classifying the co-exposure to social isolation (SI) and peripheral work position (PWP) (see method section).

## Results

Increased risks of DP were seen for most combinations of social isolation and peripheral work position in all four strata when compared to socially integrated individuals who were well attached to the labor market (Fig 1). The HRs were particularly high among men and women aged 20–39 who were socially isolated and in a peripheral work position. The HRs were not statistically significant for women aged 20–39 with partial social isolation and in a non-peripheral work position, nor for women and men aged 40–64 with no social isolation and in a partly peripheral work position.

After controlling for country of birth and long-standing illness, the associations largely remained the same, but the estimates were on a lower level (Table 3) compared with the estimates (HRs) shown in Fig 1 where no control for country of birth and long-standing illness was included. Women and men aged 20–39 with a high degree of social isolation and a peripheral work position had HRs for DP that were around 4 and 6 times higher than those of the reference group, respectively (HR 4.07; CI 2.99–5.56 for women and HR 5.70; CI 3.74–8.69 for men). Some of the intermediate combinations of social isolation and peripheral work position also showed substantially increased risk estimates. For example, young men with no social isolation and in a partly peripheral work position had a HR for future DP of 3.77; CI 2.50–5.67. A few deviances from the general pattern could also be noted. For women aged 40–64 with no social isolation and in a peripheral work position, the risk estimate for DP was very similar to that of women in the same age group with social isolation and in a non-peripheral work position (category 7 and category 3 in Table 3, HR 1.25 CI; 0.98–1.58 and HR 1.28 CI; 1.04–1.58 respectively).

Table 4 shows that the HRs for DP were considerably increased for women and men in the two age groups also when the joint exposure was based on the dichotomized indices.

The excess risk due to interaction was analyzed by two models. In the first model, where age at interview (one-year intervals) and year of interview were controlled for, statistically significant interaction effects between social isolation and peripheral work position were found among women in both age groups (aged 20–39 S = 1.62; CI 1.07–2.44 aged 40–64 S = 1.92; CI 1.01–3.64), but not among men, although there was a non-significant tendency in the same direction among the younger men (S = 1.53; CI 0.93–2.51). In the second model, where country of birth and long-standing illness were added as potential confounders, the interaction effects were below statistical significance in all strata (Table 4).

## Discussion

The aim of the study was to examine different combinations of social isolation and peripheral work position as predictors of DP and to estimate the potential interaction effects between the two. Increased risks of DP were found for different combinations of social isolation and peripheral work position among women and men in both age groups. The HRs were particularly high among men and women aged 20–39 with a high degree of social isolation and who were in a peripheral work position.

The high relative risks of DP among young people may reflect the fact that individuals in this period of life are particularly vulnerable when they find themselves outside of employment norms or lacking good social relations [1]. For young men, the negative effect of being in a peripheral work position was strongest, while for young women both indices seemed to contribute equally. Also, among the older men, the strongest associations involved peripheral work position. Despite the comparatively high level of equality between men and women in Sweden, these results may reflect that, for the men, it is still worse to “fail” as a breadwinner.

Our main interest has been to explore potential interaction (synergy) effects between social isolation and peripheral work position relative to future disability pension, but there are also possibilities of antagonistic effects between the two exposure variables [28]. The analysis of interaction effects additionally showed a stronger effect of the combination of social isolation and peripheral work position compared to each factor occurring alone, for both age groups of women, but not among men. However, as the excess risks related to the interaction did not remain significant after controlling for long-standing illness at baseline and country of birth, part of the interaction effect from being both socially isolated and in a peripheral work position may be explained by previous illness or country of birth.

On the whole, the present study indicated that participation in working life acts against future DP, especially among young adults. The finding that social isolation and peripheral work position may reinforce each other in a negative spiral has also been shown in previous research [20,21,25]. The mechanisms seem somewhat different in relation to sex and to age. For women, but not for men, the results were in accordance with the supposed mutually reinforcing effects between peripheral work position and social isolation, which were outlined in the introduction. Few deviances from the general pattern could be noted and there were no obvious suggestions that a negative effects from peripheral work position can be buffered by a high degree of social intergration in private life. As far as we know, this is the first study which has analyzed the interaction effects of the combination of social isolation and peripheral work position on the risks for DP. Further studies are necessary in order to consolidate the present results and to better establish the impact of age and sex differences on such results.

## Strengths and Limitations

This prospective study was based on representative samples from the Swedish population, utilizing high quality interview data from a large number of interviews, produced with satisfactory response rates. An additional strength was the long follow-up period and that the incidence of DP was obtained from high quality national registers. Finally, it was also possible to control for selected confounding factors such as self-reported long-standing illness at the start of follow-up. There are different ways of estimating interaction effects. The main advantages of the method used in this study is that the variables did not have to be interval scales and that the estimates could be interpreted close to the risk estimates used (the Hazard Ratios).

A limitation was that social isolation and peripheral work position were measured at one point in time and that any changes in the exposure variables that occurred after the interview could not be taken into account. Unfortunately the used data sources did not have more detailed information on some aspects of interest. Information on employment status such as type of employment contract–fixed or any form of temporary contract would have been useful to estimate the degree of precariousness employment. More detailed information on social networks would most likely improve the measurement of social isolation. Another problem is that some individuals may have used the possibility to go into early old age retirement before the age of 64. In that group there may be an overrepresentation of individuals with health reasons for early retirement related to their life situation including social aspects and labor market position. The share among individuals 60–64 that went into early retirement during our study period increased from about 4% in the early 1990s to about 14% in 2008.

## Conclusions

Individuals who were socially isolated as well as in a peripheral work position had an increased risk of future DP. The interaction effect that was suggested among women indicate that social isolation and peripheral work position may reinforce each other in regard to DP. A practical conclusion for politicians and companies is that increasing unemployment and increasing employment insecurity as well as delayed family building and lack of time for face-to-face relationships–circumstances which are becoming prevalent among young people in Sweden and other countries–are all factors that may further increase the risk of DP and permanent exclusion from working life.
